# Ocular Infiltration as the Only Relapse Sign of Atypical Lymphoma Under Chemotherapy: A Case Report

**DOI:** 10.7759/cureus.4049

**Published:** 2019-02-11

**Authors:** Lujain Alqurashi, Abdullah Alqahtani

**Affiliations:** 1 Ophthalmology, King Saud Bin Abdulaziz University for Health Sciences, Jeddah, SAU; 2 Oncology, King Saud Bin Abdulaziz University for Health Sciences, Jeddah, SAU

**Keywords:** vitreoretinal lymphoma, orbital lymphoma, recurrence, intraocular natural killer/t-cell lymphoma, diagnostic vitrectomy, smile regimen

## Abstract

Among the variants of non-Hodgkin’s lymphoma (NHL), peripheral T-cell lymphomas (PTCLs) are the least occurring. An aggressive subtype is the extranodal natural killer/T-cell lymphoma (ENKTCL) which commonly affects the nasal cavity. Ocular complications of the disease could arise due to anatomical adjacency yet it is seldom reported. We present the case of a 42-year-old male diagnosed with ENKTCL, nasal type stage IV-B with central nervous system involvement. The patient underwent three cycles of chemotherapy to which there was a complete response until relapse occurred in the form of progressive vision loss and right optic nerve infiltration. A diagnostic vitrectomy was performed, and vitreous fluid flow cytometry revealed the presence of natural killer (NK) cells. Ocular manifestations are rarely reported in the literature, yet this could be crucial to follow up on. A regular ophthalmological examination should be warranted for all cases of ENKTCL with the aim of detecting recurrence and prevention of vision loss.

## Introduction

Among the many variants of non-Hodgkin's lymphoma (NHL), peripheral T-cell lymphomas (PTCLs) are of the least occurring. A highly aggressive subtype is the extranodal natural killer/T-cell lymphoma (ENKTCL) which according to the World Health Organization (WHO) classification of lymphoid neoplasms, is its own recognized entity, representing 10% of PTCLs worldwide [1]. Typical sites of the disease include the nasal area and paranasal sinuses, hence the nomenclature ‘nasal type’, although non-nasal sites have been reported to be affected as well. It has been noted that the presence of widespread disease and the disease presence in non-typical sites is usually accompanied by a poor prognosis. Another factor that denotes an unfavorable outcome is the common affiliation with the circulating Epstein-Barr virus (EBV) deoxyribonucleic acid (DNA) [1]. Ocular lymphomas are usually of putative B-cell origin while cases of orbital NKTCL are seldom reported [2]. Ocular complications of the disease could arise due to the anatomical adjacency [3]. These complications range from uveitis and vitritis to macular holes and retinal detachments [2]. The presence of ocular complications has been reported to reduce the median overall survival from 12.5 months to four months; hence, a less desirable outcome [2]. Accordingly, ophthalmological examinations are critical in detecting and treating such cases. Ocular complaints should entail an immediate referral and thorough evaluation with aim of salvaging the vision and early detection of disease recurrence. It has been advised that initial and follow-up ocular assessments should be made mandatory in all ENKTL cases [3].

## Case presentation

**Figure 1:**
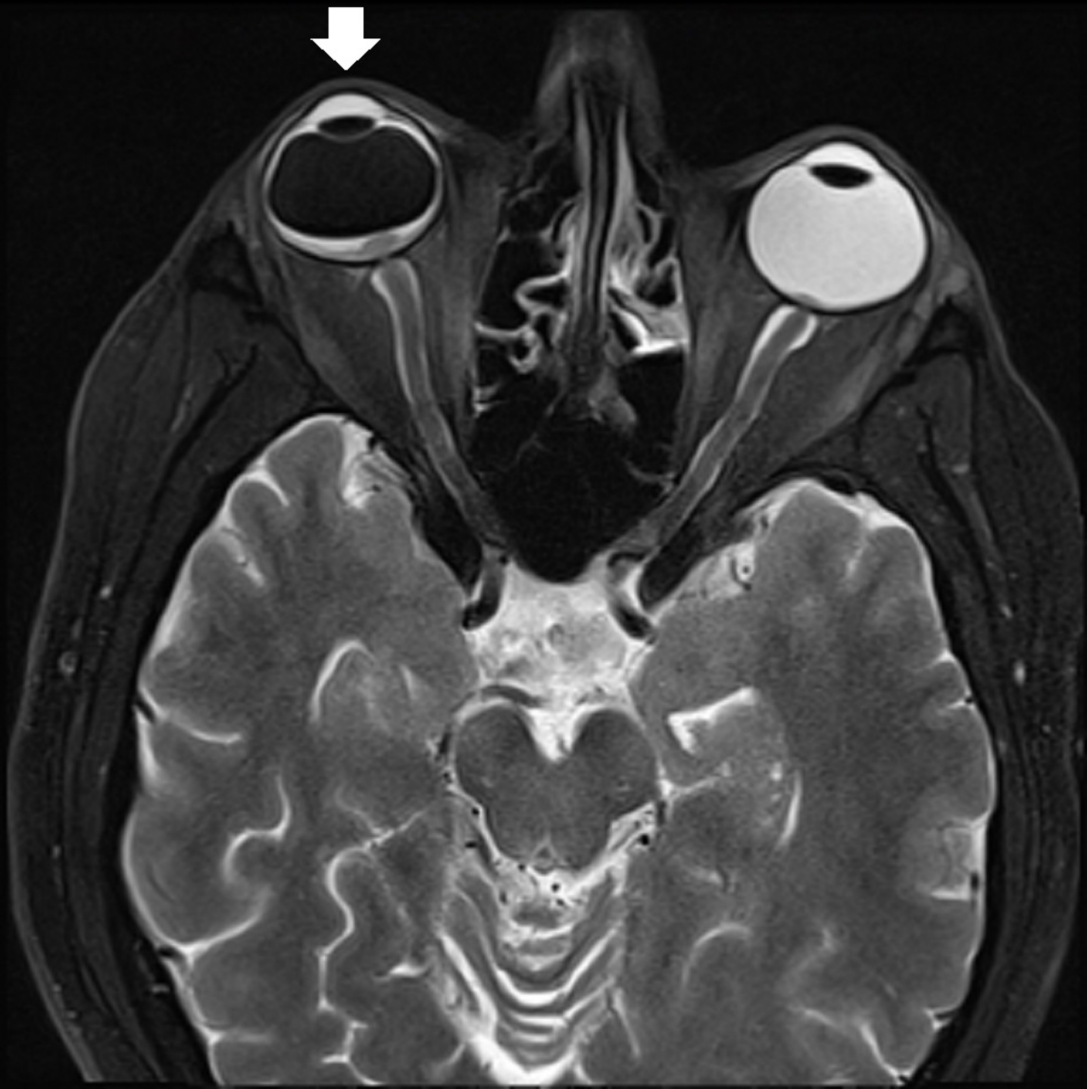
An orbital magnetic resonance imaging (MRI) revealed evidence of an enhancing retinal lesion centered on the optic disc with diffuse restriction consistent with lymphomatous infiltration; proptosis of the right eye was also noted

**Figure 2:**
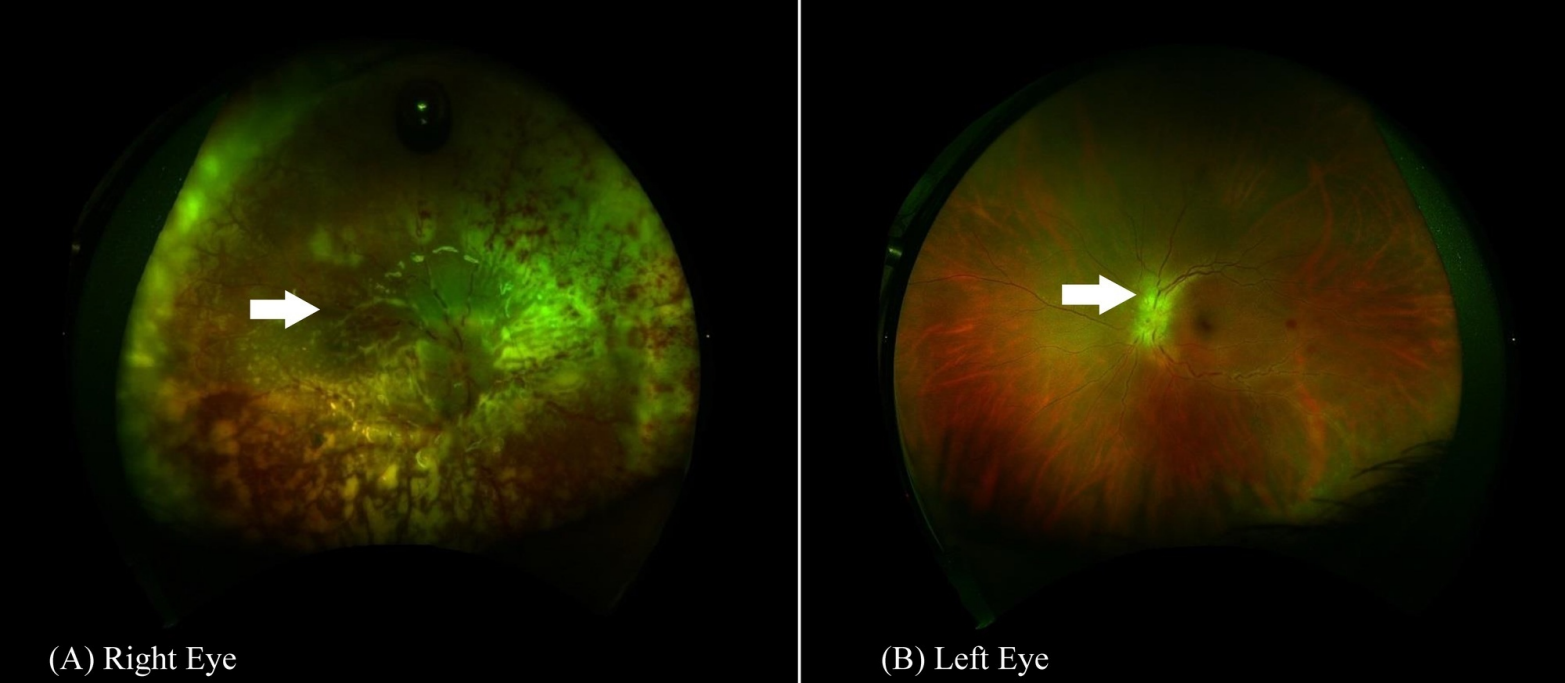
Fundus photography of both eyes performed postoperatively Wide angle fundus photography of the right eye (A) showing white-colored tissue subretina representing inflammatous infiltration of the retina and infiltration of the optic disc. In the left eye (B), however, the white-colored tissue over the optic disc represents the lymphomatous infiltration.

A 42-year-old male was referred to his ophthalmologist regarding the complaint of sudden blurriness of vision in his right eye. He was diagnosed five months ago with a case of ENKTCL, nasal type, stage IV-B with testicular and central nervous system involvement with 23% natural killer (NK) cells on cerebrospinal fluid (CSF) flow cytometry. He was on an active therapeutic plan consisting of cycles of chemotherapy (i.e., SMILE protocol: dexamethasone, methotrexate, ifosfamide, L-asparaginase, etoposide), to which there was a complete response in the form of negative flow cytometry and computed tomography (CT) of the chest, abdomen, and pelvis. Five months after the initial diagnosis, prior to the fourth cycle of treatment, a complaint of blurriness of right eye vision was made. Brain CT was done aiming to rule out ocular involvement, which turned out to be unremarkable. At the time of the complaint, visual acuity was 20/30 -2 in the right eye as opposed to 20/20 in the left eye. The right eye showed relative afferent pupillary defect. Extraocular muscle movements were competent in both eyes. On examination of the anterior chamber, +1 cells were visualized in the right eye only. On dilated fundus examination, there was vitritis in the right eye which obscured the vision. Left eye examination was insignificant. B scan ocular ultrasonography revealed retinal detachment in the right eye. An optic CT revealed vitritis in the right eye; an impression of disease infiltration of the eye was made. The therapeutic plan was a diagnostic vitrectomy followed by systemic therapy. A pars plana vitrectomy was made and the vitreous specimen was sampled revealing the presence of 10% viable lymphoid cells expressing CD2 and CD56 on immunohistochemistry stain. An orbital magnetic resonance imaging (MRI) was ordered revealing evidence of an enhancing retinal lesion centered on the optic disc with diffuse restriction consistent with lymphomatous infiltration; minimal proptosis of the right eye was noted (Figure 1). Fundus photography of both eyes was performed postoperatively with the retina flat under the silicon in the right eye as seen in Figure 2A and minimal disease involvement of the left eye which further confirmed the diagnosis (Figure 2B). In addition, CSF flow cytometry revealed immunophenotypic evidence of disease (75% mature T cells and 20% NK cells expressing CD2, CD16 and CD56). Follow-up examination revealed 6/9 vision in the left eye, as opposed to no light perception in the right eye. The patient received 35 Gray units of radiotherapy to the optic apparatus and posterior globe of both eyes and the entire cranium down to the third cervical spine region. Post-radiotherapy MRI revealed interval regression of the disease in the form of resolution of bilateral optic disc infiltrative nodule. Despite medical efforts, the patient’s condition deteriorated, and he passed away.

## Discussion

Due to the rarity of its occurrence, atypical forms, and a tendency to mimic many diseases, the diagnosis of orbital NKTCL is rendered challenging [4-5]. Many cases in the literature have initially been misdiagnosed as another disease till the malignancy was proven later on, thus hindering an early intervention. In a study done by Alqahtani et al., atypical presentations of vitreoretinal lymphomas were noted to have more virulent outcomes and it was advised that clinicians be aware of such presentations as prognosis is inversely affected by a late disease discovery [4]. In a large cohort of ENKTCL with ocular involvement reported by Woog et al., eight cases were reported in which four had initially concurrent systemic and ocular involvement at presentation, five with concurrent sinonasal involvement and three with sole ocular involvement [6]. In another study by Jiménez-Pérez et al., an increment in the mortality rate was observed, in cases of nasal NKTCL, from 55% to 80% depending on the presence of secondary ocular involvement. It is crucial to keep a low threshold for diagnostic intervention whenever suspicion arises in cases of atypical presentations with lymphoma in order not to delay the diagnosis as the prognosis greatly relies on early intervention [7].

## Conclusions

Ocular manifestations, although rarely reported in the literature, could be a very important basis in the treatment response monitoring in cases of NKTCL. A regular ophthalmological examination should be warranted for these cases with the aim of detecting recurrence and prevention of vision loss. Clinical awareness of any atypical ocular presentations in NKTCL cases should necessitate thorough workup in order not to hinder an early discovery of disease relapse.
